# NADPH oxidase as a therapeutic target in Alzheimer's disease

**DOI:** 10.1186/1471-2202-9-S2-S8

**Published:** 2008-12-03

**Authors:** Michelle L Block

**Affiliations:** 1 Department of Anatomy and Neurobiology, Virginia Commonwealth University Medical Campus, Richmond, VA 23298, USA

## Abstract

At present, available treatments for Alzheimer's disease (AD) are largely unable to halt disease progression. Microglia, the resident macrophages in the brain, are strongly implicated in the pathology and progressively degenerative nature of AD. Specifically, microglia are activated in response to both β amyloid (Aβ) and neuronal damage, and can become a chronic source of neurotoxic cytokines and reactive oxygen species (ROS). NADPH oxidase is a multi-subunit enzyme complex responsible for the production of both extracellular and intracellular ROS by microglia. Importantly, NADPH oxidase expression is upregulated in AD and is an essential component of microglia-mediated Aβ neurotoxicity. Activation of microglial NADPH oxidase causes neurotoxicity through two mechanisms: 1) extracellular ROS produced by microglia are directly toxic to neurons; 2) intracellular ROS function as a signaling mechanism in microglia to amplify the production of several pro-inflammatory and neurotoxic cytokines (for example, tumor necrosis factor-α, prostaglandin E2, and interleukin-1β). The following review describes how targeting NADPH oxidase can reduce a broad spectrum of toxic factors (for example, cytokines, ROS, and reactive nitrogen species) to result in inhibition of neuronal damage from two triggers of deleterious microglial activation (Aβ and neuron damage), offering hope in halting the progression of AD.

## Introduction

Alzheimer's disease (AD) affects more than 4 million people in the United States [1] and an estimated 27 million are affected worldwide [2]. Increasing with the aging population, the number of affected individuals is expected to triple by 2050 [1]. AD is a devastating disease, aggressively eroding the memory and cognitive function of patients across time, while robbing families, friends, and caretakers of their loved ones. At present, available treatments are unable to halt the progression of AD, making the identification of novel treatments for prevention and neuroprotection a pressing scientific concern. The following review centers on the role of microglia, the resident innate immune cells in the brain, and how this cell type contributes to progressive neuron damage, the role of NADPH oxidase in deleterious microglial activation, and how we may be able to target this key neurotoxic process to halt neurodegenerative diseases such as AD.

## Microglia and inflammation-mediated neurodegeneration

**Figure 1:**
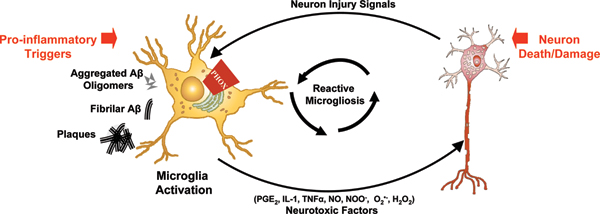
Microglia-mediated neuron damage. Microglia activation has been implicated in the progressive nature of Alzheimer's disease. Microglia can become deleteriously activated in response to disease-specific stimuli (amyloid-β (Aβ) oligomers, Aβ fibrils, and senile plaques) to produce a catalogue of factors, such as reactive oxygen species and cytokines that are toxic to neurons. In addition to disease-specific pro-inflammatory stimuli, neuronal damage/death can also activate microglia to produce these toxic factors. This continual and self-perpetuating cycle of neuronal damage/death followed by microglial activation is commonly referred to as reactive microgliosis and may be an underlying mechanism of the progressive nature of diverse neurodegenerative diseases, including Alzheimer's disease. Although all forms of Aβ have yet to be tested in detail, NADPH oxidase (also called phagocytic oxidase (PHOX)) has been implicated as a key mechanism through which microglia damage neurons in response to Aβ and neuron damage/death. This figure is modified from Block *et al*. [3]. NO, nitric oxide; PGE_2_, prostaglandin E2; TNF, tumor necrosis factor.

There is a wealth of evidence demonstrating that microglia, the resident innate immune cells in the brain, can become deleterious and damage neurons [3,4]. This process is implicated as an underlying mechanism in diverse neurodegenerative diseases, including AD [3,4]. While microglial function is beneficial and mandatory for normal central nervous system functioning, microglia become toxic to neurons when they are over-activated and unregulated [4]. Microglia are activated in response to specific stimuli to produce pro-inflammatory factors (for example, tumor necrosis factor (TNF)α, prostaglandin E2 (PGE_2_), and interferon-γ) and reactive oxygen species (for example, ^•^NO, H_2_O_2_, O_2_^•-^, ONOO^-^/ONOOH), which are toxic to neurons [4,5]. Microglia actively monitor the brain and can become activated to cause neuron damage in response to two categories of stimuli. First, microglia can identify pro-inflammatory triggers, such as β-amyloid (Aβ), resulting in activation, the production of toxic factors, and neuron death/damage (Figure 1). Second, the microglial response to neuronal damage can also become toxic (Figure 1) [5]. Current evidence demonstrates that the microglial response to neuronal damage can be long-lived, self-perpetuating, and toxic to neurons [3,5,6] (Figure 1). This repeating cycle of the neurotoxic activation of microglia in response to neuron injury is commonly referred to as reactive microgliosis (Figure 1). In fact, it has been proposed that deleterious microglial activation may be propagated and potentially amplified throughout multiple neurodegenerative diseases, including AD [3].

## Alzheimer's disease, microglial activation, and oxidative stress

Pathological diagnosis of AD is characterized by the identification of insoluble extracellular plaques containing Aβ and intraneuronal neurofibrillary tangles in the cortical region of the brain. The premise of microglia over-activation in AD has been supported by analysis of *post mortem *brains from AD patients, where there is clear evidence of microglial activation in association with lesions of senile, amyloid plaques and neurofibrillary tangles [7,8]. In fact, microglial activation occurs early in AD development, before neuropil damage, supporting a contributing role of microglia in disease pathology [9]. Further, the microglial response to Aβ [10,11] and the senile plaques [12,13] includes the production of toxic factors. For example, TNFα [14], nitric oxide [15], and superoxide [11] are produced by microglia in response to Aβ. Epidemiological studies have further supported the role of inflammation in AD, where results have shown a decreased incidence and severity of AD in patient populations treated with nonsteroidal anti-inflammatory drugs (NSAIDs) [16-18]. Thus, evidence supports the presence of microglial activation in AD and that this may contribute to the pathology of the disease [19], where Aβ is hypothesized to be a key element in the pro-inflammatory mechanism.

The amyloid hypothesis has been modified over time, but the premise has remained that Aβ has a causative role in AD pathology [20], both through direct toxicity to neurons [21], and by causing neuronal damage through microglial activation [20]. Aβ(1–40) and Aβ(1–42) are the predominant forms of Aβ in the brain, and have been implicated as active participants in the mechanism of AD progression. Aβ(1–42) has been labeled as the more toxic of the two prevalent Aβ peptides, and toxicity is dependent upon the aggregation state of Aβ, and the mode of toxicity (that is, directly toxic to neurons or microglia-mediated neurotoxicity) [20,22,23]. The aggregation state of Aβ required to cause microglia activation and neuron damage is disputed. For example, small, soluble oligomers of Aβ have been shown to activate microglia [24], but the majority of studies suggest that the larger, insoluble Aβ aggregates, and fibrillized Aβ, are the more potent stimuli [25,26]. Undoubtedly, Aβ has been shown to both recruit and activate microglia [8,14,27], suggesting a critical role in AD progression.

In addition to microglial activation and cytokine production, oxidative stress is closely associated with AD [28], where early AD pathology shows evidence of oxidative damage [29-31], indicating a potential role in disease pathogenesis. The detailed mechanisms of how oxidative stress causes AD are complex and poorly understood, but AD-specific patterns of oxidative stress have been identified. For example, localized increases in carbonylated-, 4-hydroxynonenal-, and 3-nitrotyrosine-modified proteins have been reported in hippocampus and parietal cortex of AD patient brains, demonstrating disease specific modifications due to reactive nitrogen species [32]. In fact, it has been repeatedly demonstrated that increased levels of nitrated proteins are present in AD brains [33-36], including tau nitration [37], which indicates that an increase in reactive nitrogen species affects AD pathology. Additionally, protein oxidation is known to induce protein aggregation, a process that has been hypothesized as a critical mechanism contributing to tau tangle formation [31], Aβ aggregation, and senile plaque formation [38]. Importantly, Aβ is also clearly indicated as a source of oxidative stress [39], as Aβ activates microglia to produce extracellular superoxide [11,40]. Thus, while there may be several sources and mechanisms, there is increasing support that oxidative stress contributes to AD pathology, and that microglia and Aβ may play an instigating role.

## NADPH oxidase, oxidative stress, and neuronal damage

NADPH oxidase is a multi-subunit enzyme complex that is activated during host defense in phagocytes, such as microglia, to catalyze the production of superoxide from oxygen. NADPH oxidase is a member of the NOX gene family, also called NOX2 and phagocytic oxidase (PHOX). The NADPH oxidase enzyme complex consists of the membrane bound cytochrome b558 (p22^PHOX ^and the enzymatic subunit, gp91^PHOX^), several cytosolic proteins (p47^PHOX^, p67^PHOX^, and p40^PHOX^), and the Rac G-protein [41,42]. NADPH oxidase is activated when the cytosolic subunits are phosphorylated and Rac is activated in the cytosol, resulting in their translocation to the membrane and formation of the active NADPH oxidase complex with cytochrome b558 [41,42]. The end product of the enzyme is superoxide.

Recently, NADPH oxidase has been associated with neurodegenerative disorders and related complications. For example, NADPH oxidase is activated in brains from AD patients [43] and is upregulated in Parkinson's disease [44]. Interestingly, reactive oxygen species (ROS) from NADPH oxidase have also been shown to mediate Aβ-induced cerebrovascular dysfunction [45]. It is not surprising that several stimuli activate NADPH oxidase in microglia to cause neuron damage, including Aβ [11,46], amyloid precursor protein [47], rotenone [48], air pollution [49], paraquat [50], substance P [51], and α-synuclein [52]. Further, NADPH oxidase has been shown to play a role in how reactive microgliosis (the microglial response to neuron damage) causes additional neuron damage [53]. Thus, evidence supports that NADPH oxidase may be a common pathway of microglia-mediated neuronal damage.

The NOX family of proteins is expressed on diverse cell types and NADPH oxidase is present in microglia, neurons, and astrocytes. However, NADPH oxidase is present in lower amounts in cells that are not from the myeloid lineage (for example, neurons and astrocytes). Additionally, astrocytes and neurons do not express several receptors that recognize and respond to stimuli that activate NADPH oxidase in microglia, including MAC1 and subsets of scavenger receptors. Further, while NADPH oxidase can be activated in these alternative cell types, the resulting production of ROS is significantly lower than that of microglia and other cells specialized for innate immunity. Notably, a study using lipopolysaccharide (LPS) as a stimulus of microglial activation showed that only NADPH oxidase from microglia, and not astrocytes and neurons, caused the NADPH oxidase-mediated neuron damage [54]. Microglia derived from NADPH oxidase knockout mice failed to produce extracellular superoxide, expressed reduced pro-inflammatory profiles, and showed significantly less intracellular ROS [54]. Thus, microglia are the predominant source of NADPH oxidase-derived ROS and targeting microglial NADPH oxidase may be an ideal approach to attenuate deleterious activation [55].

In addition to damaging neurons through the production of extracellular superoxide, NADPH oxidase also impacts neuron survival by regulating the microglial pro-inflammatory response [56]. The PHOX-ROS pathway refers to the signaling mechanism resulting from the increase in intracellular ROS in phagocytes as a response to NADPH oxidase activation. The increase in intracellular ROS in phagocytes, such as microglia, includes the production of several radicals, including the superoxide anion, hydroxyl radical, lipid hydroperoxides, and their by-products (for example, H_2_O_2_) [57]. Normal cellular function and metabolism results in the production of intracellular ROS from multiple cellular sources, including mitochondrial electron transport, xanthine oxidase, peroxisomes, and the endoplasmic reticulum [57]. However, NADPH oxidase contributes to a large proportion of intracellular ROS in phagocytes in response to an immunological stimulus, where approximately 50% of the LPS-induced intracellular ROS increase in microglia is due to NADPH oxidase [54]. The relative contribution of NADPH oxidase to the increase in microglial intracellular ROS is stimulus dependent, as substance P-induced intracellular ROS is largely dependent upon NADPH oxidase [51].

NADPH oxidase enhances pro-inflammatory gene expression through several downstream signaling molecules, for example, protein kinase C, mitogen-activated protein kinase activation, and nuclear factor-κB (NFκB) [58-60]. Thus, NADPH oxidase initiates an intracellular ROS signaling pathway [61] that can activate microglia and amplify the production of multiple pro-inflammatory cytokines, such as TNFα [54] or PGE_2 _[62]. In fact, several triggers of NADPH oxidase activation in microglia amplify pro-inflammatory signaling. For example, gangliosides are shown to activate microglia, where the production of interleukin (IL)-1β, TNF-α, and inducible nitric oxide synthase (iNOS) are attenuated by the addition of the NADPH oxidase inhibitor diphenyleneiodonium (DPI) [63]. Furthermore, both DPI and catalase (an enzyme that catalyzes the decomposition of H_2_O_2 _to water and oxygen) were shown to suppress LPS-induced expression of cytokines (IL-1, IL-6, and TNFα), iNOS, mitogen-activated protein kinases, and NFκB phosphorylation [64]. In addition to cytokine production, NADPH oxidase has also been shown to mediate the morphological changes associated with microglial activation [54]. Thus, intracellular ROS play an essential role in the regulation of general microglial activation.

Additionally, NADPH oxidase is reported to prime microglia for enhanced sensitivity to additional stimuli. For example, rotenone [65] and neuronal death [66] have been shown to prime microglia through NADPH oxidase to synergistically enhance cytokine production and neurotoxicity upon additional exposure to LPS. Thus, not only does activation of NADPH oxidase amplify the microglial pro-inflammatory response, it also changes this response to additional stimuli, allowing a robust pro-inflammatory response to previously innocuous stimuli. Taken together, microglial NADPH oxidase activation, and the production of ROS, have been implicated as critical regulators of microglia-mediated neurotoxicity and represent ideal therapeutic targets.

## Anti-inflammatory therapy and Alzheimer's disease

Given the strong evidence for the presence of microglial activation, oxidative stress, and pro-inflammatory factors in AD [67], there has been a keen interest in the potential therapeutic utility of anti-inflammatory drugs [68,69]. Unfortunately, while many epidemiological and animal studies have supported the use of this approach, clinical trials have not been successful.

The bulk of previous anti-inflammatory studies have focused predominantly on NSAIDs [70]. Epidemiological studies have shown that patients taking anti-inflammatory medicine for rheumatoid arthritis were six times less likely to develop AD [71]. Other epidemiological studies have also shown that diets high in anti-oxidants may decrease the risk of developing AD [72]. Additionally, a separate epidemiological study shows that taking NSAIDs for at least one month is associated with lower probability of AD [73]. Interestingly, NSAIDs may also protect against cognitive decline in the elderly without AD diagnosis [74]. However, only two pilot trials have shown some promise [73,75], and the majority of clinical results for NSAIDS have been disappointing. One explanation proposed for the success of NSAIDs in both animal and epidemiological studies, yet failure in clinical trails, is that NSAIDs may be able to prevent disease, rather than treat symptoms [73]. Another hypothesis is that the anti-inflammatory compounds tested may not be focusing on the most deleterious and damaging consequences of microglial activation.

## Neuroprotection and NADPH oxidase inhibitors

**Figure 2:**
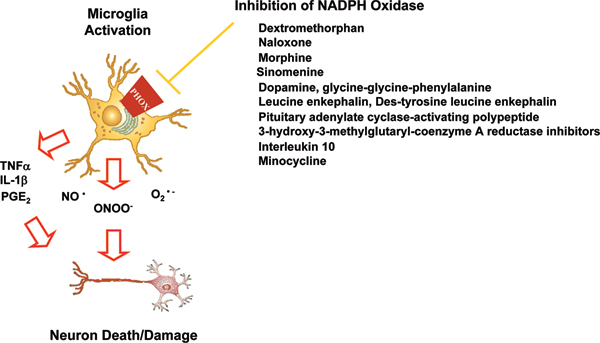
NADPH oxidase inhibition targets deleterious microglial activation. Increasing evidence points to NADPH oxidase (also called phagocytic oxidase (PHOX)) as a critical mechanism of microglia-mediated neuron damage. Traditional anti-inflammatory approaches focus on specific downstream targets, such as prostaglandin E2 (PGE_2_). However, targeting NADPH oxidase inhibits the global pro-inflammatory response further upstream in the process of neurotoxic microglial activation and is able to inhibit a broad spectrum of cytokines, nitric oxide, and reactive oxygen species to confer neuroprotection. At present, small molecules, peptides, anti-inflammatory cytokines, and an antibiotic have been identified that inhibit microglial NADPH oxidase and are neuroprotective. Further research is warranted to discover the mechanisms through which these seemingly diverse compounds work and to identify more specific inhibitors of this key neurotoxic pathway. This figure is modified from Zhang *et al*. [82]. IL, interleukin; TNF, tumor necrosis factor.

Traditional NSAIDs, such as indomethacin and aspirin, target a single pro-inflammatory factor, such as PGE_2_, where the target is the downstream result of the toxic microglial pro-inflammatory response. However, microglia produce several pro-inflammatory factors and ROS [4], where successful inhibition of microglia-mediated toxicity will likely require the attenuation of a broad spectrum of factors. To address this problem, our approach has been to focus on regulating the microglial function upstream, before the response becomes toxic, and to focus on what we believe to be the most detrimental component of microglial activation, NADPH oxidase activation (Figure 2).

Recently, several peptides (pituitary adenylate cyclase-activating polypeptide, dynorphin, glycine-glycine-phenylalanine, leucine enkephalin, and des-tyrosine leucine enkephalin) [76-78], antibiotics (minocycline) [79], and small molecules (dextromethorphin, statins, naloxone, and sinolimine) [77,80,81] have been identified that inhibit NADPH oxidase and are neuroprotective (Figure 2). For example, dextromethorphan (DXM) illustrates the broad therapeutic utility of NADPH oxidase inhibitors [82]. DXM is a noncompetitive N-methyl-d-aspartate (NMDA) receptor antagonist that is widely and commercially used as an antitussive agent, and is neuroprotective through inhibition of microglial activation and NADPH oxidase activation [83,84]. In addition, DXM has been tested for a variety of conditions, and has shown activity in ameliorating pain [85] and as a neuroprotectant against focal ischemia [86]. In particular, DXM is noted to have anti-inflammatory effects [87], and is reported to protect against neuron damage through the inhibition of microglial activation in methamphetamine-induced neurotoxicity [88], and in studies of *in vitro *[83,89] and *in vivo *[90] models of Parkinson's disease. While the NMDA inhibitor dizocilpine maleate fails to inhibit NADPH oxidase, and is not neuroprotective [84], a recent report showed that the NMDA receptor antagonist memantine may also be neuroprotective through microglia inhibition [91]. While it is clear that DXM works through the inhibition of NADPH oxidase [83,84], and inhibition of the NMDA receptor does not always result in neuroprotection [89], the role of the NMDA receptor in DXM inhibition of NADPH oxidase remains unclear. Notably, DXM, statins, and naloxone have a history of safe therapeutic use, supporting that inhibition of NADPH oxidase is safe. For the purpose of targeting this enzyme complex for the therapeutic treatment of neurodegenerative diseases, future efforts will need to focus on the identification of both the detailed mechanisms of NADPH oxidase inhibition and developing more specific inhibitors.

## Conclusion

Increasing evidence points to NADPH oxidase as a critical component of deleterious microglial activation. Key components of AD pathology, for example, Aβ fibrils and plaques, can serve as triggers of microglial NADPH oxidase activation and associated neuron damage. Additionally, NADPH oxidase has been implicated in the neurotoxic response of microglia to neuronal damage. Activation of microglial NADPH oxidase causes neuron damage through the production of neurotoxic extracellular ROS, enhancement of the global microglial pro-inflammatory response, and the priming of microglia to have a heightened sensitivity to previously innocuous stimuli. In this manner, inhibiting NADPH oxidase may target the progressive cycle of deleterious microglial activation that fuels progressive neurotoxicity. At present, while 'old drugs' have been identified that are neuroprotective through their effects on NADPH oxidase, and may be useful for current therapy, future research must focus on elucidating the detailed molecular mechanisms of the diverse list of NADPH oxidase inhibitors and then move forward with developing more specific, potent, and safe agents.

## List of abbreviations used

Aβ: beta amyloid; AD: Alzheimer's disease; DPI: diphenyleneiodonium; DXM: dextromethorphan; IL: interleukin; iNOS: inducible nitric oxide synthase; NMDA: N-methyl-d-aspartate; NSAID: nonsteroidal anti-inflammatory drug; PGE_2_: prostaglandin E2; PHOX: phagocytic oxidase; ROS: reactive oxygen species; TNF: tumor necrosis factor.

## Competing interests

The author declares that they have no competing interests.
